# Phospholipase D Family Member 4 Regulates Microglial Phagocytosis and Remyelination via the AKT Pathway in a Cuprizone‐Induced Multiple Sclerosis Mouse Model

**DOI:** 10.1111/cns.70111

**Published:** 2024-11-15

**Authors:** Ran Sun, Tengyun Ma, Zheng Zhao, Yan Gao, Juan Feng, Xue Yang

**Affiliations:** ^1^ Department of Neurology Shengjing Hospital of China Medical University Shenyang People's Republic of China; ^2^ Department of Emergency Medicine Shengjing Hospital of China Medical University Shenyang People's Republic of China

**Keywords:** AKT, cuprizone, multiple sclerosis, phagocytosis, phospholipase D family member 4, remyelination

## Abstract

**Aims:**

Remyelination is an endogenous repair process that is often deficient in multiple sclerosis (MS). Stimulation of remyelination is thought to help limit the progression of MS. This study aimed to investigate the expression pattern and function of a microglial phagocytosis‐related gene, phospholipase D family member 4 (PLD4), in a cuprizone (CPZ)‐induced MS mouse model.

**Methods:**

The extent of remyelination was assessed using LFB staining. Myelin phagocytosis assay was used to investigate the effect of Pld4 on microglial phagocytic activity.

**Results:**

Pld4 was upregulated in the corpus callosum during demyelination and remyelination. AAV9‐mediated Pld4 deficiency impaired remyelination and reduced the number of Olig2‐positive cells. In the corpus callosum of Pld4‐deficient mice, the microglial phagocytosis marker MAC2 was reduced, accompanied by inhibition of TrkA/AKT signaling. Similarly, the phagocytosis assay showed that Pld4 knockdown significantly inhibited myelin debris phagocytosis by BV2 cells. The AKT activator SC79 reversed the Pld4 deficiency‐induced inhibition of microglial phagocytic activity and rescued the impaired remyelination in Pld4‐deficient mice.

**Conclusion:**

PLD4 is upregulated in CPZ‐induced MS and modulates microglial phagocytosis and remyelination via the AKT pathway. Our findings provide experimental evidence for a better understanding of the molecular mechanism of MS.

## Graphical abstract

PLD4 is upregulated in CPZ‐induced MS and modulates microglial phagocytosis and remyelination via the AKT pathway. PLD4 deficiency resulted in inhibition of microglial phagocytosis of myelin debris and impaired remyelination in the corpus callosum.

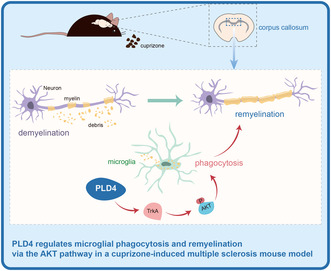

**Abbreviations**
AAV: adeno‐associated virus
CPZ: cuprizone
DEGs: differentially expressed genes
EAE: experimental autoimmune encephalomyelitis
Hck: hematopoietic cell kinase
Iba1: allograft inflammatory factor 1
IF: immunofluorescence
IHC: immunochemistry
LFB: Luxol fast blue
MBP: myelin basic protein
MS: multiple sclerosis
Olig2: oligodendrocyte transcription factor 2
OPCs: oligodendrocyte precursor cells
PLD4: phospholipase D family member 4
Sh3bp1: SH3‐domain binding protein 1
TNFR2: TNF receptor 2
TrkA: tropomyosin receptor kinase A

## 1 Introduction

Multiple sclerosis (MS) is an autoimmune disease affecting the central nervous system, characterized by demyelinating lesions of nerve fibers (loss of myelin and myelin‐producing oligodendrocytes). Typical clinical manifestations include muscle weakness, sensory and cognitive impairment, as well as fatigue [1]. The myelin sheath, which surrounds the neuron axons, is essential for nerve protection and insulation. Damage to myelin impairs the propagation of action potentials and leads to axonal damage [2]. The etiology of MS is complex and largely unknown. Although currently approved disease‐modifying therapies with immunomodulatory drugs for MS relieve symptoms and improve life quality, it is difficult to reverse the progression of myelin degeneration, and the financial burden and adverse effects of long‐term medication should also be considered [3].

Once demyelination has occurred, oligodendrocyte precursor cells (OPCs) are recruited to the damaged sites where they proliferate and differentiate into myelin‐producing oligodendrocytes, contributing to remyelination in MS lesions [4, 5]. Remyelination is an endogenous repair process; however, remyelination is frequently inadequate in MS. Therefore, stimulating remyelination is thought to help limit the progression of MS. The cuprizone (CPZ) diet is often used to induce MS in mice. CPZ is a copper chelator that selectively causes oligodendrocyte loss, leading to extensive demyelination in the mouse brain [6, 7]. Since new oligodendrocytes are not destroyed by CPZ, ~75%, ~85%, and ~100% spontaneous remyelination is observed within the first 3 weeks after CPZ withdrawal [8]. Therefore, the CPZ diet model is suitable for investigating the underlying mechanisms of the demyelination and remyelination phases in MS. Microglia‐mediated phagocytosis of myelin debris is thought to be a prerequisite for remyelination [9, 10], and inefficient clearance of myelin debris impedes remyelination processes involving OPC recruitment and maturation [11].

Phospholipase D family member 4 (PLD4) is a single‐stranded exonuclease that regulates endosomal nucleic acid sensing [12]. PLD4 has been identified as a susceptibility gene for autoimmune diseases such as systemic lupus erythematosus and systemic sclerosis [13, 14] and as a biomarker for Alzheimer's disease [15]. PLD4 is abundant in microglia and is involved in microglial phagocytosis [16]. Chiba et al. observed delayed myelination in Pld4‐knockout mice, suggesting a potential role for Pld4 in myelination [17]. Interestingly, PLD4 expression in chronic active and chronic lesions of MS patients is significantly lower than that in normal brain white matter controls [18], suggesting a link between PLD4 loss and MS progression. Taken together, these findings help us to establish a potential link between PLD4 and microglial phagocytosis of myelin debris in MS.

## 2 Methods

### 2.1 Animal Study

Male C57BL/6 mice aged 8–10 weeks were used for modeling. The animal experiment procedure was approved by the Ethics Committee of the Shengjing Hospital of China Medical University and followed the ARRIVE guidelines. As previously described, cuprizone intoxication was induced by feeding mice 0.2% w/w CPZ in standard chow powder [19, 20]. The experimental time points were: demyelination (5 weeks of CPZ feeding; CPZ5) and remyelination (5 weeks of CPZ feeding followed by 1 week of CPZ withdrawal; CPZ5 + 1). Four weeks prior to CPZ feeding, in vivo adeno‐associated virus (AAV, serotype 9) delivery of PLD4 shRNA (under the microglia‐specific F4/80 promoter) to the corpus callosum was performed using the following stereotaxic coordinates: anterior to posterior −2, medial to lateral ±0.5, and dorsal to ventral −1.2 (mm relative to Bregma, with reference to the Fifth edition of the Paxinos and Franklin Mouse Brain Atlas and previous research) [21, 22]. Each mouse received an injection of 2 μL AAV9 (5 × 10^8^ genome copies/μL) as previously reported [23, 24]. The microglia‐specific PLD4 shRNA AAV9 was packaged by WZ Biosciences Inc. (Jinan, China) using a miR‐30‐shRNA interference AAV vector.

### 2.2 Neurologic Function Evaluation

Neurologic deficits were scored according to the criteria in previous research, ranging from 0 (normal neurologic functions) to 5 (severe neurologic function loss) [25]. The pole test was performed to evaluate the climbing ability as previously described [26].

### 2.3 Luxol Fast Blue (LFB) Staining

Mouse brains were coronally sectioned to 5‐μm slices. To assess the extent of demyelination and remyelination, the dewaxed and rehydrated brain slides were incubated with LFB solution (CatLog S3382, Sigma‐Aldrich, MO, USA) at 57°C for 4 h. For color development, the slides were incubated with 0.05% Li_2_CO_3_ solution (CAS 554‐13‐2, Sinopharm Chemical, Shanghai, China) until the white matter turned dark blue. The slides were then washed, restained with LFB solution for 1 min, and observed under a light microscope.

### 2.4 Real‐Time PCR

RNA was extracted by the phenol–chloroform method. Reverse transcription of RNA was conducted using reverse transcriptase and RNase inhibitor (CatLog D7160L, Beyotime Institute of Biotechnology; CatLog B600478, Sangon Biotech; Shanghai, China). The mRNA expression was assessed by real‐time PCR. Real‐time PCR was performed using SYBR‐containing PCR master mix (CatLog PC1150 and SY1020, Solarbio Ltd. Beijing, China) on an Exicycler 96 PCR system (Bioneer, Daejeon, Korea) under standard PCR conditions (95°C for 5 min; then 40 cycles of 95°C for 10 s, 60°C for 10 s, and 72°C for 15 s). The primer used were: *Gapdh* (Forward primer: TGTTCCTACCCCCAATGTGTCCGTC, Reverse primer: CTGGTCCTCAGTGTAGCCCAAGATG), *Pld4* (Forward primer: TGGTGCCCAGATACGACA, Reverse primer: AGGGATGGAAGCGGTTGA), *Fcgr1* (Forward primer: TTCAGATTCGGAGGTCG, Reverse primer: AGCACTGGCGTGGTAAA), *Scarb1* (Forward primer: ATCTGGTGGACAAATGGAAC, Reverse primer: TGAAGCGATACGTGGGAAT), *Sh3bp1* (Forward primer: AGGTGCCCTCAAGTCCTATCT, Reverse primer: GTTTACATCCTGCTCCTCTGC), and *Hck* (Forward primer: GCCAAGTGCCAATCAGA, Reverse primer: AGCCCTCCACAAACCCT).

### 2.5 Preparation of Lysates and Immunoblot Analysis

BV2 cells or corpus callosum tissues were lysed on ice in RIPA buffer supplemented with PMSF (CatLog P0100, Solarbio). Supernatants were collected after centrifugation. Protein concentrations were measured and equalized by mixing with loading buffer. The proteins were then resolved by SDS‐PAGE and transferred to PVDF membranes. The membranes were then incubated in blocking solution (CatLog SW3010, Solarbio) at room temperature for 1 h. After washing, the membranes were probed with the following primary and secondary antibodies: PLD4 (Affinity Company DF4294; RRID: AB_2836645), GAPDH (ProteinTech Group 60,004‐1‐Ig; RRID: AB_2107436), tropomyosin receptor kinase A (TrkA; Affinity DF6822; RRID: AB_2838782), AKT (Affinity AF6261; RRID: AB_2835121), p‐AKT Ser473 (Affinity AF0016; RRID: AB_2810275), and HRP‐conjugated secondary antibodies (Solarbio SE134 and SE131; RRID: AB_2797593 and AB_2797595). Finally, protein bands were visualized using an enhanced chemiluminescence technique. Full unedited immunoblots are available in the Supporting Information.

### 2.6 Immunofluorescence (IF) and Immunochemistry (IHC) Staining

Brain sections or fixed cell slides were incubated with primary antibodies against PLD4 (Affinity DF4294; RRID: AB_2836645), allograft inflammatory factor 1 (Iba1; Abcam ab283319; RRID: AB_2924797), MAC2 (also known as galectin‐3; Affinity AF0164; RRID: AB_2833357), myelin basic protein (MBP; Affinity AF4085; RRID: AB_2835364), and oligodendrocyte transcription factor 2 (Olig2; ABclonal Biotechnology A12814; RRID: AB_2759654) at 4°C overnight. After washing, brain sections or cell slides were incubated with secondary antibodies conjugated to fluorescent dye or horse radish peroxidase. For IHC staining, diaminobenzidine was utilized for color development. Finally, the fluorescence microscope and light microscope were used for microphotography.

### 2.7 Phagocytosis Assay

The corpus callosum of C57BL/6 mice was mechanically homogenized and centrifuged at 1000 *g* for 10 min, and the supernatants were collected for the myelin debris phagocytosis assay as previously described [27, 28]. After 2 h of serum starvation, mouse microglial BV2 cells were incubated with 15 μg/mL debris for 24 h in the presence or absence of 100 ng/mL LPS. The percentage of myelin‐containing (MBP‐positive) cells was calculated to assess phagocytic activity.

### 2.8 RNA‐Seq

After 2 h of serum starvation, mouse microglial BV2 cells were incubated with 15 μg/mL debris for 24 h. The cells were then collected, lysed, and total RNA was extracted. After RNA enrichment and library preparation, the samples were subjected to sequencing on the Illumina NovaSeq 6000 platform. After removing the adaptors and low‐quality reads, the clean reads were mapped to the mouse genome with Hisat2 and then subjected to further analysis. Differentially expressed genes (DEGs) were defined as those with log2FC ≥ 0.5 or ≤ −0.5 and adjusted *p* < 0.05 by using the R package DESeq2.

### 2.9 Statistics

All data were tested for normality using the Shapiro–Wilk test. The data that passed the normality test are presented as mean ± SD and were analyzed using the Student's *t*‐test (for two groups) or one‐way ANOVA with post hoc test (for three or more groups). The data that did not pass the normality test are presented as median and interquartile range and were analyzed using the Kruskal–Wallis test with post hoc test. The level of significance is indicated as: *p < 0.05, **p < 0.01, and ***p < 0.001.

## 3 Results

### 3.1 Pld4 Is Upregulated in the Corpus Callosum During Demyelination and Remyelination

**FIGURE 1:**
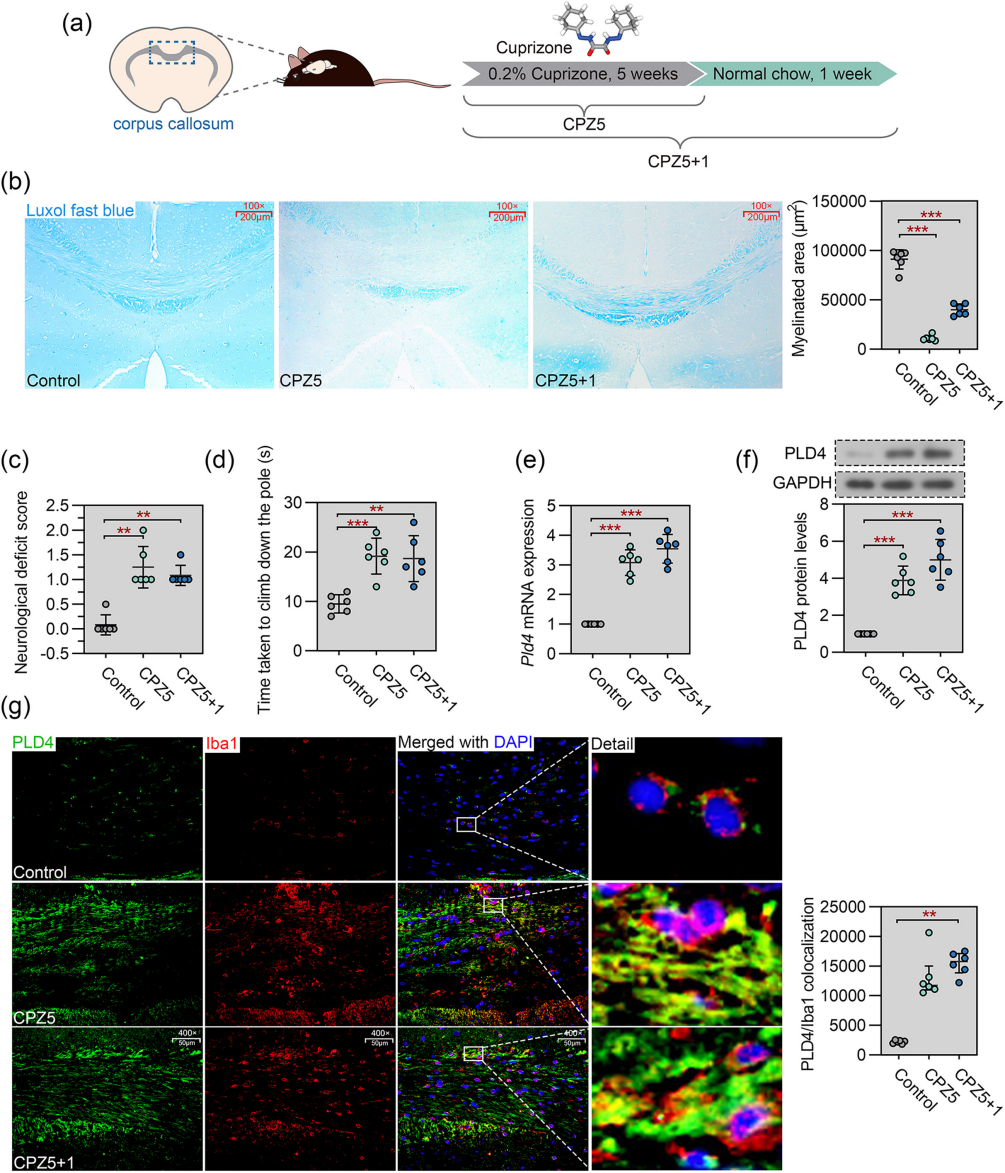
Pld4 is upregulated in the corpus callosum during demyelination and remyelination. Mice were fed a 0.2% w/w cuprizone (CPZ) diet for 5 weeks for demyelination (CPZ5), followed by 1 week of normal chow for remyelination (CPZ5 + 1). Control mice received normal chow. (a) Schematic representation of the experimental design. The image of 3D structure of cuprizone was downloaded from PubChem. (b) Luxol fast blue (LFB) histochemistry was used to assess demyelination and remyelination in the corpus callosum. The right panel shows the quantification of the myelinated area. Magnification: 100×; scale bar: 200 μm. (c) The neurologic deficit score of the mice. (d) Time taken to climb down the pole in the pole test. (e) Real‐time PCR was used to evaluate the expression of Pld4 mRNA in the corpus callosum. (f) Immunoblot was performed to evaluate the level of PLD4 protein in the corpus callosum. (g) Left panel: Representative images of double immunofluorescence (IF) staining of PLD4 (green) and Iba1 (red) in the corpus callosum. Magnification: 400×; scale bar: 50 μm. Right panel: Quantification of PLD4/Iba1 colocalization. *N* = 6 in each group. Data are expressed as mean ± SD. **p < 0.01, and ***p < 0.001 compared with Control.

Mice were intoxicated with CPZ for 5 weeks (CPZ5) and then fed normal chow for 1 week (CPZ5 + 1). Control mice received normal chow (Figure 1a). We first examined CPZ‐induced pathologic changes in the corpus callosum at different experimental time points. As revealed by LFB staining, CPZ intoxication for 5 weeks caused a robust loss of myelin in the corpus callosum. After cessation of CPZ feeding, remyelination was observed (Figure 1b). To determine whether there was an association between myelin pathology and neurologic function, we assessed neurologic deficit scores and performed the pole test to assess motor coordination in mice. As shown in Figure 1c,d, CPZ‐fed mice had higher neurologic deficit scores and took more time to climb down the pole than control mice, and these results could not be completely reversed by 1 week of normal chow recovery.

CPZ intoxication also resulted in a marked increase in Pld4 expression, both at mRNA and protein levels, and this increase was not eliminated by 1 week of normal chow (Figure 1e,f). The colocalization of PLD4 and Iba1 was also increased in demyelinated mice and even continued to be slightly increased in recovering mice (Figure 1g), indicating a sustained accumulation of PLD4 in microglial cells in the corpus callosum after CPZ intoxication.

### 3.2 Pld4 Deficiency Impairs Remyelination in the Corpus Callosum

**FIGURE 2:**
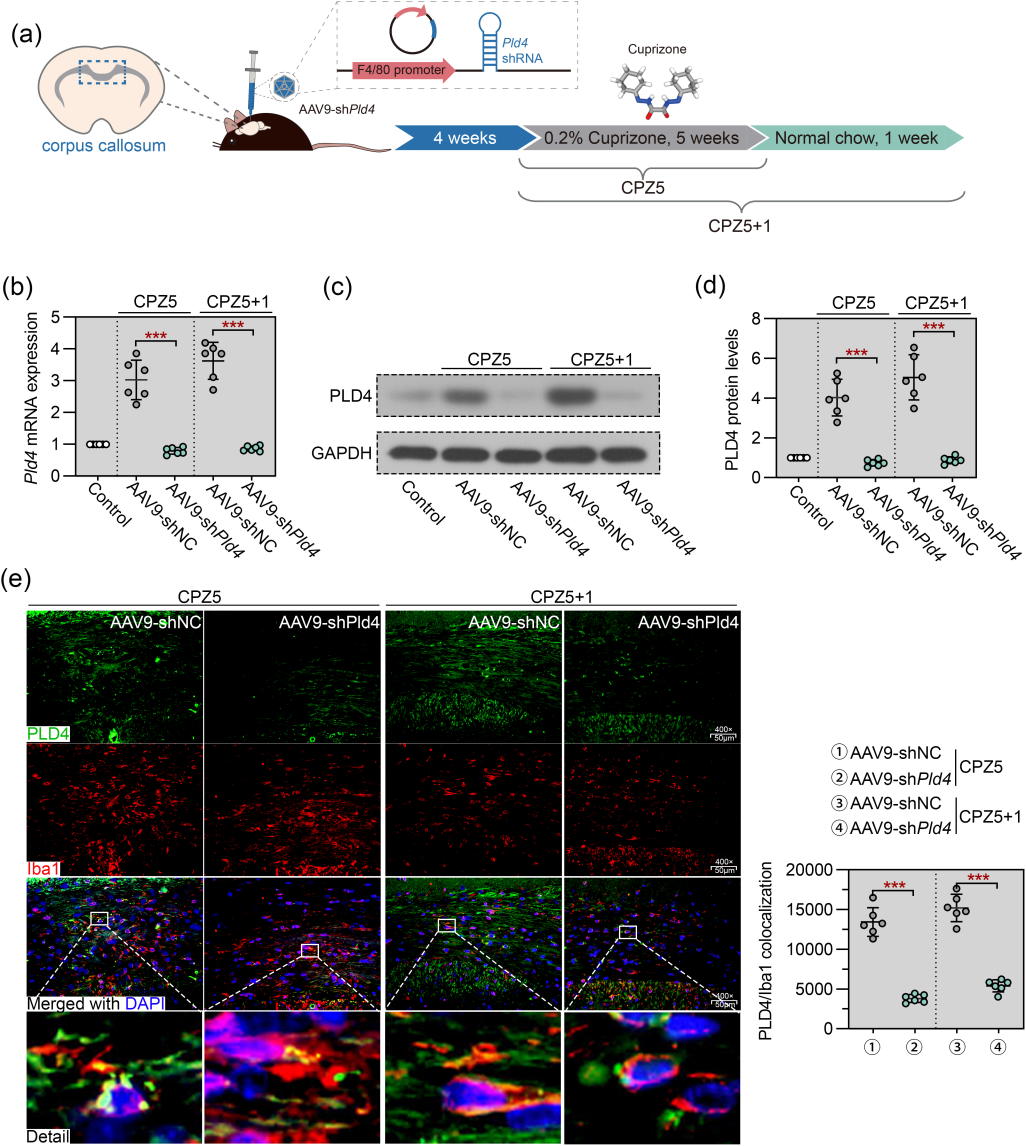
Pld4 was knocked down in microglial cells in the corpus callosum. Knockdown of Pld4 was achieved by adeno‐associated virus (AAV) delivery of Pld4 shRNA directly into the corpus callosum of mice 4 weeks before CPZ administration. (a) Schematic representation of the experimental design. The image of 3D structure of cuprizone was downloaded from PubChem. (b) Real‐time PCR was used to evaluate the expression of Pld4 mRNA in the corpus callosum. (c, d) Immunoblot was performed to evaluate the level of PLD4 protein in the corpus callosum. (e) Left panel: Representative images of double IF staining of PLD4 (green) and Iba1 (red) in the corpus callosum. Magnification: 400×; scale bar: 50 μm. Right panel: Quantification of PLD4/Iba1 colocalization. *N* = 6 in each group. Data are expressed as mean ± SD. ***p < 0.001.

Knockdown of Pld4 was achieved by injection of microglia‐specific F4/80 promoter‐driven shPld4 AAV9 into the corpus callosum 4 weeks prior to CPZ feeding (Figure 2a). As manifested by real‐time PCR and immunoblot, AAV9‐shPld4 induced a sustained knockdown of Pld4 throughout the experimental period (Figure 2b–d). Double IF staining of PLD4 and Iba1 showed that AAV9‐mediated knockdown of Pld4 reduced PLD4 expression within microglia (Figure 2e).

**FIGURE 3:**
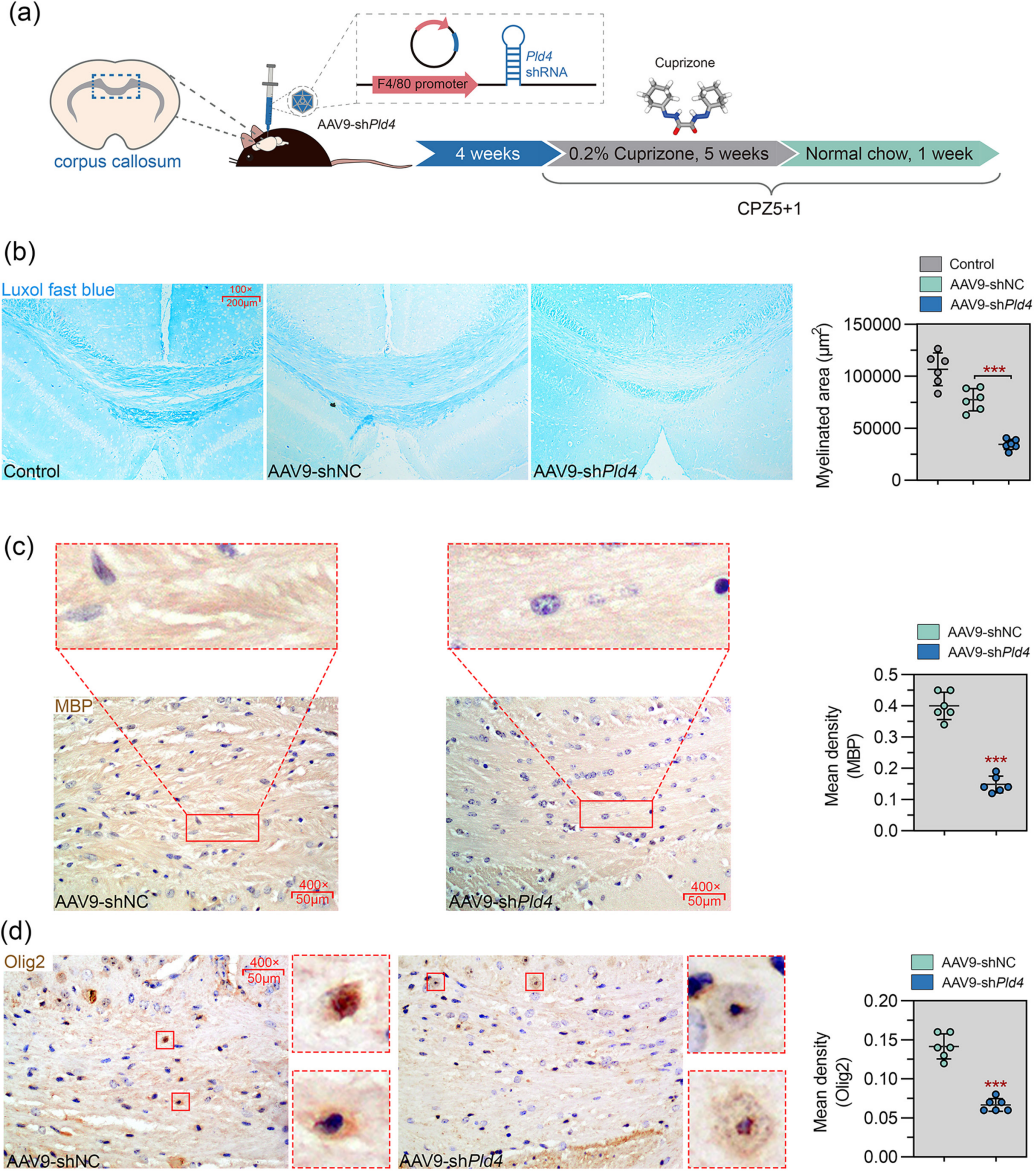
Pld4 deficiency impaired remyelination in the corpus callosum. Corpus callosum samples were collected from mice fed a CPZ diet for 5 weeks followed by a normal diet for 1 week. (a) Schematic representation of the experimental design. The image of 3D structure of cuprizone was downloaded from PubChem. (b) Left panel: Representative images of LFB histochemistry was used to assess demyelination and remyelination in the corpus callosum. Magnification: 100×; scale bar: 200 μm. Right panel: Quantification of the myelinated area. (c) Left panel: Representative images of immunohistochemistry for myelin basic protein (MBP) in the corpus callosum. Magnification: 400×; scale bar: 50 μm. Right panel: Quantification of mean density. (d) Left panel: Representative images of immunohistochemistry for oligodendrocyte transcription factor 2 (Olig2) in the corpus callosum. Magnification: 400×; scale bar: 50 μm. Right panel: Quantification of mean density. *N* = 6 in each group. Data are expressed as mean ± SD. ***p < 0.001 compared with shNC.

We next evaluated the effect of Pld4 knockdown on remyelination after CPZ withdrawal (Figure 3a). As shown in Figure 3b, after 1 week of normal chow, LFB myelin staining in the corpus callosum of shNC mice showed a moderate recovery compared to healthy control mice, whereas remyelination in the corpus callosum of Pld4‐deficient mice was markedly impaired. IHC for MBP revealed that Pld4‐deficient mice had much more pronounced myelin loss than shNC mice (Figure 3c). We then performed IHC staining for the oligodendrocyte lineage marker Olig2. We found that Pld4 knockdown significantly reduced the number of Olig2‐positive cells (Figure 3d), implying an association between Pld4 and OPC recruitment or maturation. These results suggest that the silencing of Pld4 causes remyelination defects in the corpus callosum.

### 3.3 Pld4 Deficiency Impairs Microglial Phagocytosis and TrkA/AKT Signaling in the Corpus Callosum

**FIGURE 4:**
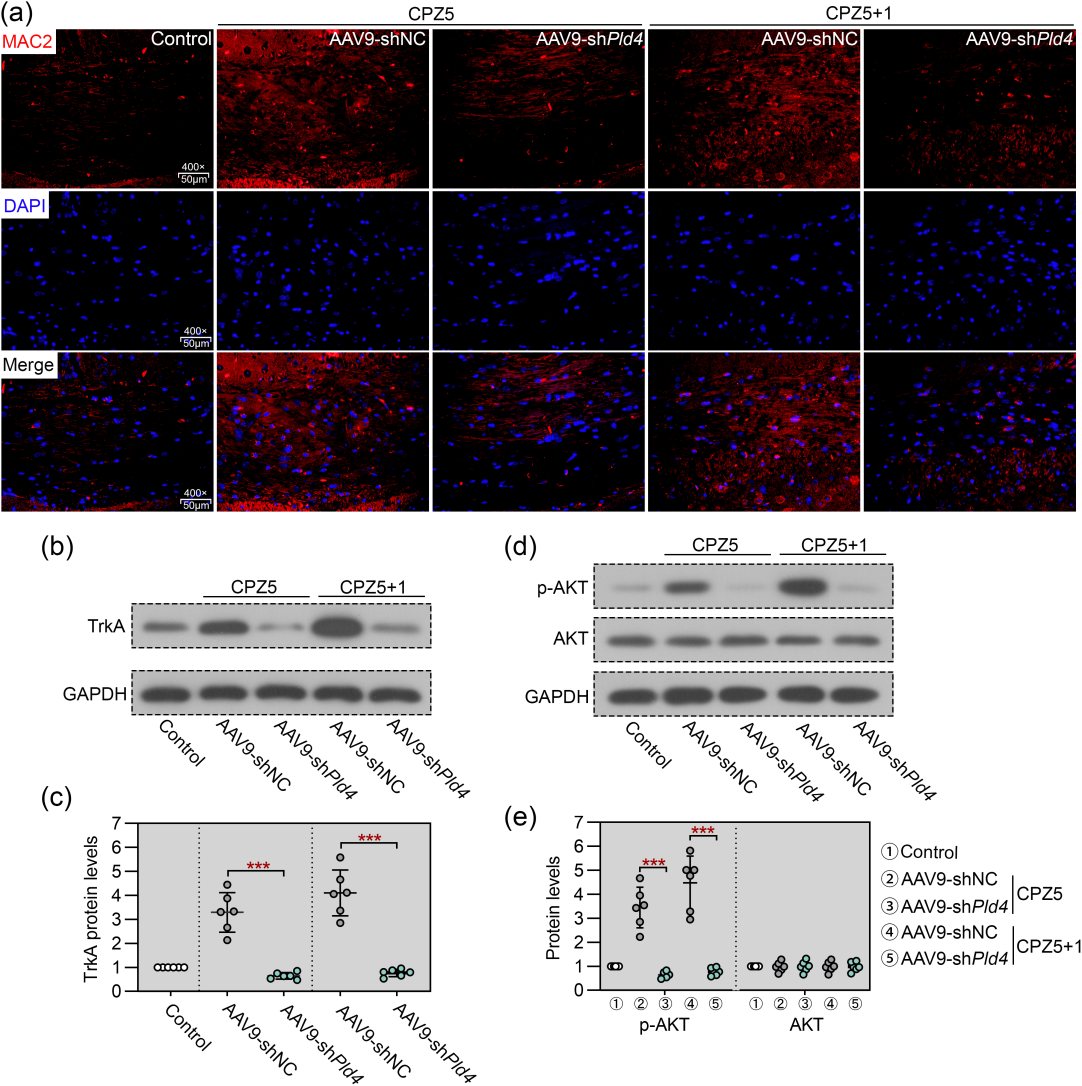
Pld4 deficiency impaired microglial phagocytosis and TrkA/AKT signaling in the corpus callosum. (a) IF staining for MAC2 (namely galectin‐3) was performed to evaluate microglial phagocytosis in the corpus callosum. Magnification: 400×; scale bar: 50 μm. (b, c) Immunoblot was performed to evaluate the level of TrkA protein in the corpus callosum. (d, e) Immunoblot was performed to evaluate the phosphorylation of AKT in the corpus callosum. *N* = 6 in each group. Data are expressed as mean ± SD. ***p < 0.001.

We next investigated whether Pld4 deficiency affects microglial phagocytic activity. The number of MAC2‐stained phagocytic microglial cells was markedly increased in response to CPZ feeding and did not return to basal levels in the remyelination phase. Pld4 Knockdown resulted in a significant reduction of MAC2‐positive cells in both the demyelination and remyelination phases (Figure 4a). As previously reported, a PLD4 downstream effector, TrkA [29], activates AKT signaling in microglia [30]. Given that activation of AKT signaling is well established in promoting microglial phagocytosis [31], we tested whether TrkA/AKT signaling is regulated by Pld4 during the demyelination and remyelination phases in MS mice. TrkA protein levels were markedly increased in the corpus callosum of mice fed CPZ for 5 weeks, and the increased TrkA levels appeared to be more pronounced after 1 week of recovery, whereas the increased TrkA was significantly inhibited in Pld4‐deficient mice during both demyelination and remyelination phases (Figure 4b,c). Consistently, the phosphorylation of AKT was also suppressed by Pld4 knockdown (Figure 4d,e). Taken together, these results suggest that Pld4 deficiency impairs microglial phagocytosis and TrkA/AKT signaling in the corpus callosum during both demyelination and remyelination.

### 3.4 Pld4 Regulates Microglial Phagocytosis of Myelin Debris Through Activation of AKT Signaling

**FIGURE 5:**
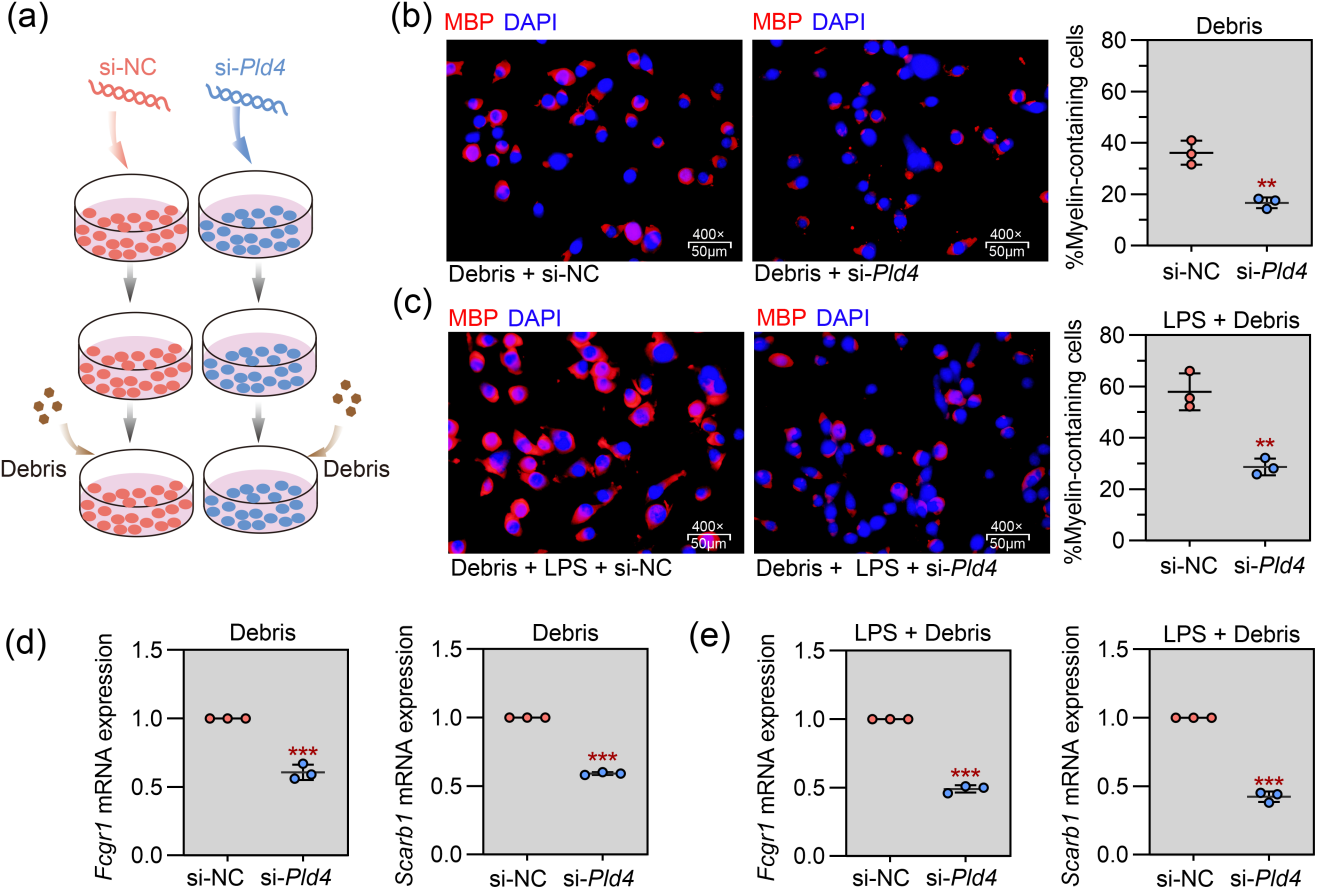
Pld4 regulates microglial phagocytosis of myelin debris in vitro. Mouse microglial BV‐2 cells were transfected with siRNA against Pld4 (si‐Pld4) to knockdown Pld4 expression. Control cells were transfected with non‐targeting siRNA (si‐NC). To evaluate the phagocytosis of myelin debris by BV‐2 cells, cells were incubated with myelin debris for 24 h in the presence or absence of LPS. (a) Schematic representation of the experimental protocol. (b, c) Left panel: Representative images of IF staining of MBP was performed to visualize myelin‐containing cells. Magnification: 400×; scale bar: 50 μm. Right panel: Quantification of the percentage of myelin‐containing cells. (d, e) Real‐time PCR was used to evaluate the mRNA expression of Fcgr1 and Scarb1 in cells. *N* = 3 in each group. Data are expressed as mean ± SD. **p < 0.01 and ***p < 0.001 compared with si‐NC group.

**FIGURE 6:**
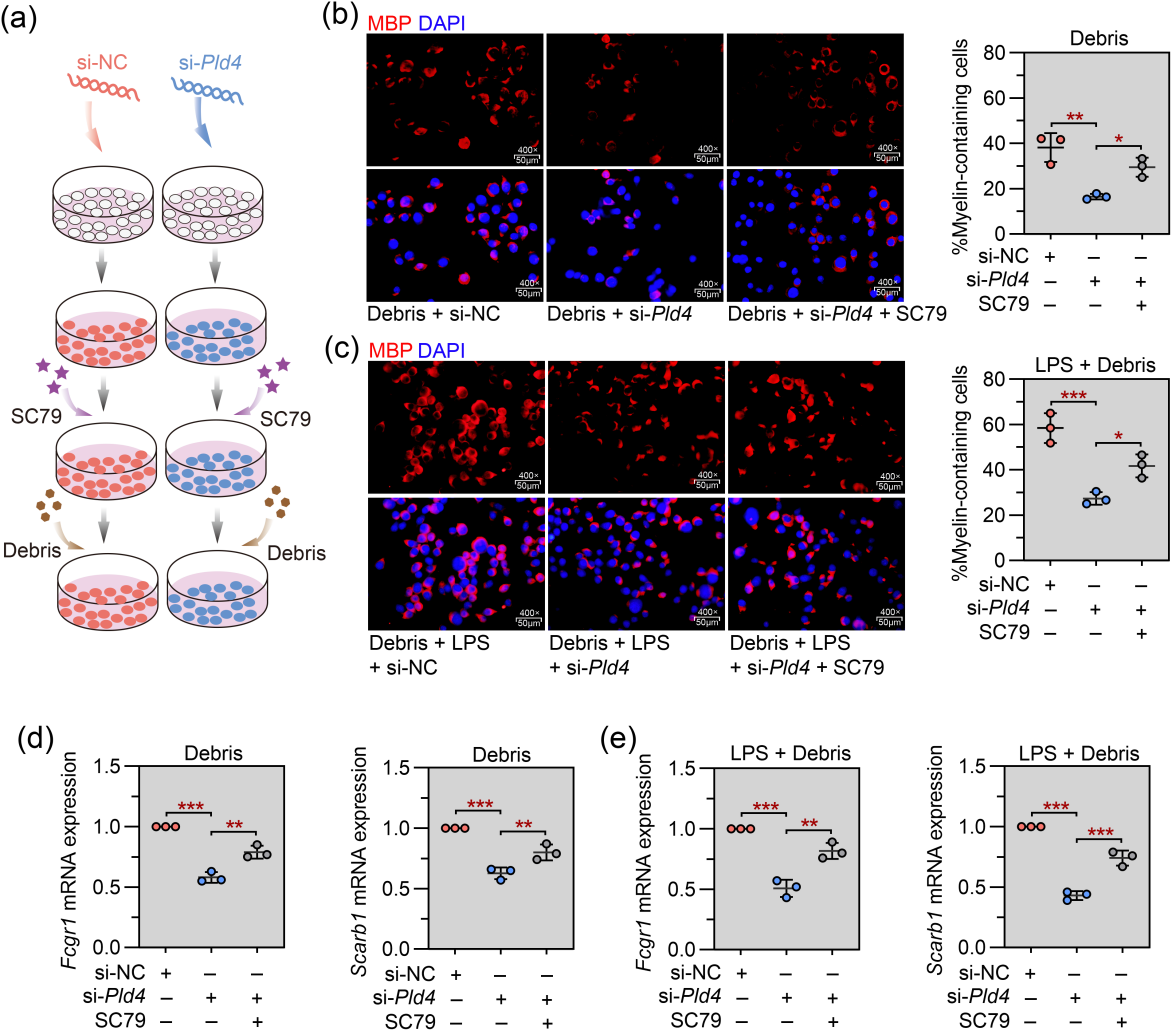
Pld4 regulates microglial phagocytosis of myelin debris through activation of AKT signaling. Two hours before incubation with myelin debris, SC79 was used to pharmacologically activate AKT signaling. (a) Schematic representation of the experimental protocol. (b, c) Left panel: Representative images of IF staining for MBP was performed to visualize myelin‐containing cells. Magnification: 400×; scale bar: 50 μm. Right panel: Quantification of the percentage of myelin‐containing cells. (d, e) Real‐time PCR was used to evaluate the mRNA expression of Fcgr1 and Scarb1 in cells. *N* = 3 in each group. Data are expressed as mean ± SD. *p < 0.05, **p < 0.01, and ***p < 0.001.

We next investigated the effect of Pld4 knockdown on the phagocytic activity of mouse microglial cells using a myelin phagocytosis assay. Pld4‐specific siRNA mediated the knockdown of Pld4 in BV2 cells (Figure 5a). As shown in Figure 5b,c, siRNA silencing of Pld4 reduced the percentage of myelin‐containing cells in the presence or absence of LPS. Additionally, Pld4 silencing significantly reduced the expression of the phagocytic marker genes Fcgr1 and Scarb1 (Figure 5d,e). Next, a small molecule activator of AKT (SC79) was introduced into the phagocytosis assay (Figure 6a). We found that SC79 increased the number of myelin‐containing cells (Figure 6b,c) and rescued the expression of phagocytic marker genes (Figure 6d,e), demonstrating that activation of the AKT signaling pathway reversed the Pld4 siRNA‐induced inhibition of microglial phagocytic activity.

### 3.5 Transcriptomic Analysis Elucidates Pld4 Knockdown‐Induced Gene Expression Changes in Phagocytic Microglia

**FIGURE 7:**
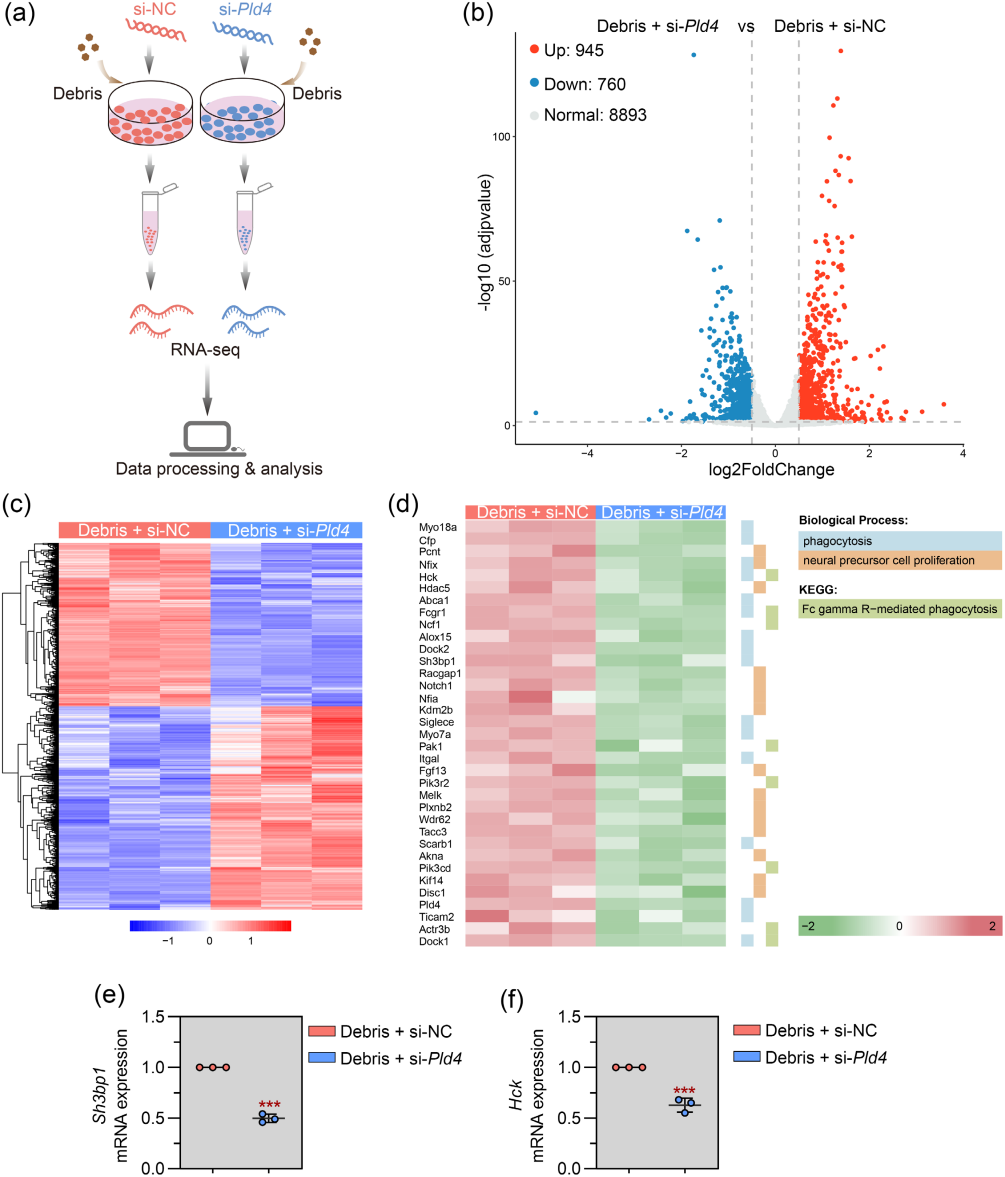
Transcriptomic analysis. (a) Schematic representation of the experimental protocol. (b) The volcano plot of the RNA‐seq data. (c) The heatmap of differentially expressed genes (DEGs). (d) The heatmap of DEGs involved in the indicated KEGG pathway and GO terms. (e, f) Real‐time PCR was used to evaluate the mRNA expression of Sh3bp1 and Hck in cells. *N* = 3 in each group. Data are expressed as mean ± SD. ***p < 0.001 compared with si‐NC group.

Transcriptomic sequencing was performed in phagocyting microglia with and without Pld4 knockdown (Figure 7a). The volcano plot showed that 945 upregulated and 760 downregulated DEGs were identified (Figure 7b). The heatmap indicated that the transcriptomic profile of phagocytic microglia is clearly separated by Pld4 knockdown (Figure 7c). Given that Pld4 knockdown reduced the phagocytic activity of microglial cells and impaired remyelination, we performed GO and KEGG analysis using downregulated DEGs, with a particular focus on phagocytosis and neural precursor cell‐related biologic processes and pathways. Figure 7d displayed the expression of the genes enriched in the indicated terms. Furthermore, real‐time PCR was used to verify the expression alterations of two phagocytosis‐related genes, SH3‐domain binding protein 1 (Sh3bp1), and hematopoietic cell kinase (Hck) (Figure 7e,f).

### 3.6 Activation of the AKT Signaling Pathway Promotes Remyelination in the Corpus Callosum by Enhancing Microglial Phagocytosis

**FIGURE 8:**
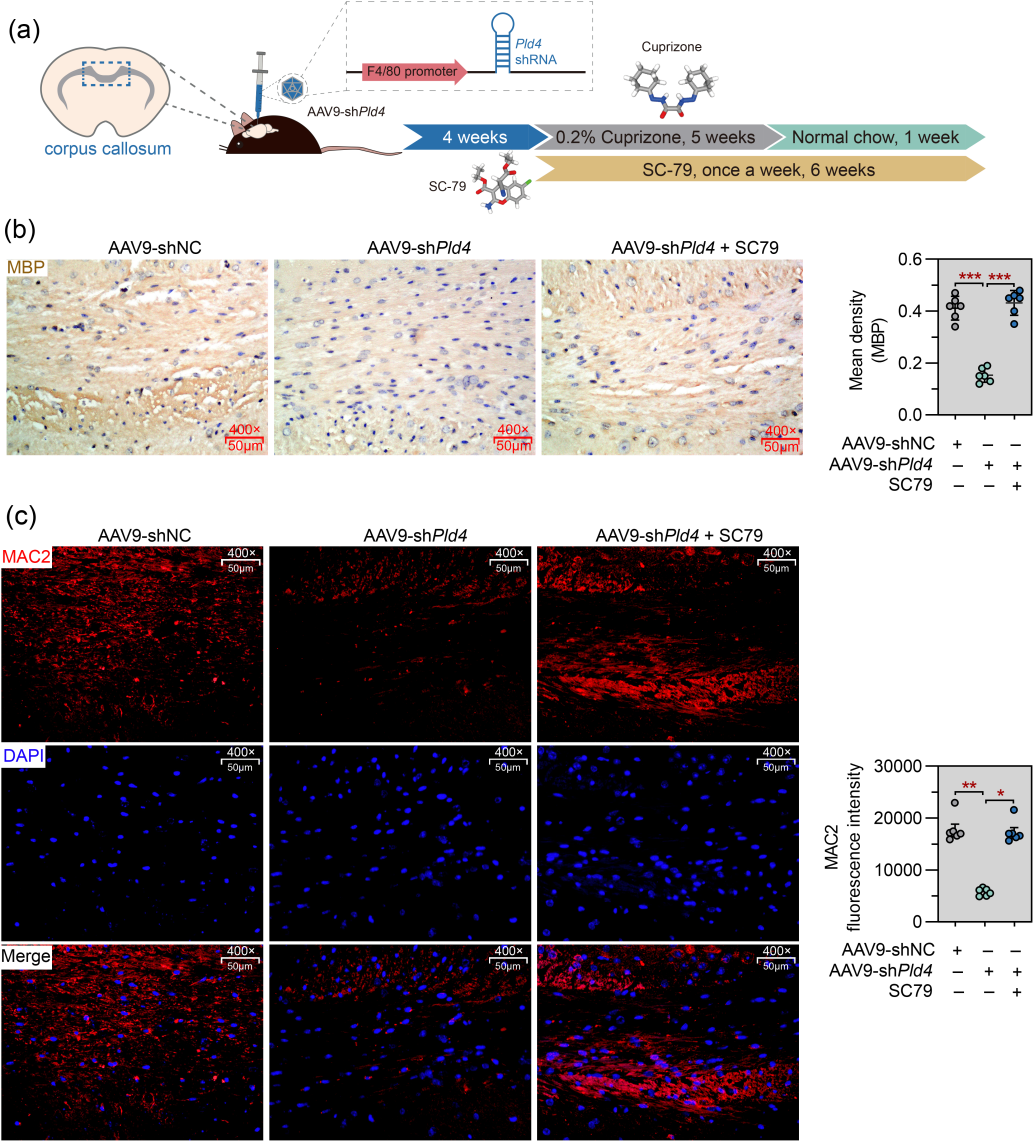
Activation of AKT signaling promotes remyelination in the corpus callosum by enhancing microglial phagocytosis. Four weeks after AAV injection, mice received intraperitoneal injection of the AKT agonist SC79 once a week during 5 weeks of CPZ diet and 1 week of normal diet. (a) Schematic representation of the experimental design. The images of 3D structure of cuprizone and SC79 were downloaded from PubChem. (b) Immunohistochemistry for MBP in the corpus callosum. Magnification: 400×; scale bar: 50 μm. (c) Left panel: Representative images of IF staining for MAC2 was performed to evaluate microglial phagocytosis in the corpus callosum. Magnification: 400×; scale bar: 50 μm. Right panel: Quantification of MAC2 fluorescence intensity. *N* = 6 in each group. Data are expressed as mean ± SD. *p < 0.05, **p < 0.01, and ***p < 0.001.

Finally, Pld4‐deficient mice received intraperitoneal injections of the AKT agonist SC79 once a week for 6 weeks (Figure 8a), to test whether AKT activation would have rescued the impaired microglial phagocytosis and remyelination in the corpus callosum. As evidenced by MBP staining, SC79 treatment predominantly promoted remyelination in the corpus callosum of Pld4‐deficient mice, which was comparable to that in shNC mice (Figure 8b). Furthermore, SC79 rescued phagocytic MAC2 expression, indicating restoration of microglial phagocytosis (Figure 8c).

## 4 Discussion

In the current study, we revealed the expression and function of Pld4 in CPZ‐intoxicated mice. To the best of our knowledge, this is the first study focusing on the role of Pld4 in MS. We found that Pld4 is significantly upregulated in microglia during demyelination and remyelination, suggesting its continuous functions in the progression of MS. Interestingly, loss of PLD4 expression was found in chronic active and chronic lesions of MS patients compared to normal brain white matter controls [18]. In autoimmune diseases such as MS, genetic factors may strongly influence the likelihood of disease progression in different individuals [32, 33]. Impaired remyelination in MS patients may be due to impaired microglial phagocytosis caused by genetic PLD4 deficiency. However, the C57BL/6 mice used in this study had basal PLD4 expression, and the increased PLD4 in response to CPZ contributes to microglial phagocytosis and myelin repair. This may explain why PLD4 expression patterns show different trends in MS patients and mouse models of MS. To determine whether PLD4 is an important genetic factor in MS, the expression and/or mutation profile of PLD4 needs to be tested in more clinical samples.

Motor deficits have been frequently reported in MS patients, but the motor deficits in animal models have not been fully characterized and conflicting results have been reported in different studies. In this study, we performed the pole test to evaluate the motor functions of mice, and we found that mice with 5 weeks of CPZ diet had obvious deficits in climbing down the pole compared with control mice, which is consistent with the findings of Dong et al. [26]. The upregulated neurologic deficit scores of CPZ‐fed mice also demonstrated CPZ intoxication of neurologic functions. Surprisingly, however, these deficits did not appear to be significantly alleviated after 1 week of CPZ withdrawal, although considerable remyelination was observed at the same time point. Similarly, Franco‐Pons et al. reported that sensorimotor reactivity deficits in CPZ‐intoxicated mice were not reversed even 6 weeks after CPZ withdrawal [34]. This may be because remyelination and neurologic improvement may not occur simultaneously, and remyelination is not sufficient to immediately alleviate neurologic dysfunction [35]. Therefore, it is necessary to further investigate the temporal relationship between histopathologic changes and neurologic functions or to use more sensitive detection indicators or behavioral experiments to observe what may be subtle neurologic functional changes during demyelination and remyelination.

The role of microglia in MS is complex and can be either disease‐promoting or protective, due to the wide range of microglial activities and their heterogeneous states. On the one hand, the pro‐inflammatory states of microglia and their persistent activation are closely associated with MS progression [36]. On the other hand, microglia also exhibit protective roles, including the removal of myelin debris and secreting regenerative factors to promote the recruitment and proliferation of OPCs and their differentiation into oligodendrocytes, thereby fostering remyelination [37]. Gao et al. reported that in an experimental autoimmune encephalomyelitis (EAE) model of MS, TNF receptor 2 (TNFR2) ablation in microglia caused early onset of EAE with increased immune cell infiltration and activation and demyelination, whereas TNFR2 ablation in monocytes/macrophages had opposite effects [38]. This study suggests that monocytes/macrophages and microglia may have completely different roles in MS. However, brain‐resident microglia and infiltrating monocyte‐derived macrophages from peripheral blood share a number of markers that make it difficult to distinguish between the two cell types when the blood–brain barrier is disrupted. In particular, the intact blood–brain barrier of CPZ‐fed mice makes it easier to study the functions of microglia without considering the involvement of peripheral macrophages. Therefore, in the present study, the suppression of phagocytosis by Pld4 knockdown in CPZ‐fed mice can be mainly attributed to its effects on microglia.

Although microglia are considered to play a dual role, protective or detrimental, in MS, it is widely accepted that microglia‐mediated phagocytosis and clearance of myelin debris can facilitate remyelination in EAE [39], which is consistent with our findings in the CPZ model. In contrast, suppression of microglia‐dependent phagocytosis of myelin debris impaired remyelination and recovery in EAE [40]. However, unlike the toxin‐induced demyelination in the CPZ model, the pathology in the EAE model is driven by self‐reactive CD4 T cells, demonstrating the involvement of microglia in the T‐cell‐mediated immune response [41]. On the one hand, microglia depletion impaired antigen presentation and T‐cell reactivation in the early phase of EAE [42], and on the other hand, microglia inhibited CD4^+^ T‐cell proliferation to suppress the secondary progression of autoimmune encephalomyelitis [43]. Therefore, the specific functions of PLD4 in the EAE model need to be further investigated.

Microglial phagocytosis consists of the engulfment of myelin debris and lysosomal processing of engulfed debris [8]. In this study, we found that Pld4‐knockdown‐impaired phagocytosis of myelin debris is associated with a reduction in the expression of Fcgr1 and Scarb1, which are thought to mediate myelin debris uptake by microglia [44, 45]. Based on these findings, we can conclude that Pld4 knockdown may affect the uptake of myelin debris, but whether Pld4 affects the lysosomal processing of myelin debris requires further investigation. In addition, it has been reported that astrocytes regulate myelin clearance by recruiting microglia in the demyelinated area in the brain of CPZ‐fed mice [46]. Therefore, whether astrocytes are involved in myelin debris clearance should also be considered in future research.

Furthermore, we conducted a high‐throughput RNA sequencing analysis to investigate the global transcriptome alterations in phagocytic microglia following Pld4 knockdown. A total of 945 upregulated and 760 downregulated genes were identified, indicating that Pld4 has a significant impact on the gene expression of phagocytic microglia. Upon GO and KEGG pathway enrichment analysis, we focused our attention on genes involved in phagocytosis and neural precursor cell proliferation. With regard to phagocytosis‐related genes, in addition to the previously determined Fcgr1 and Scarb1, which were reduced in response to Pld4 deficiency. Furthermore, Sh3bp1 and Hck were also found to be downregulated following Pld4 knockdown. Sh3bp1 is a member of the RhoGAP family, which is responsible for the inactivation of GTPase, thereby contributing to the phagocytosis, specifically the internalization, of large particles [47]. Notably, an increase in Sh3bp1 expression was observed in the corpus callosum of CPZ‐fed mouse, indicating its potential involvement in MS [48]. Hck is a crucial regulator of phagocytosis in myeloid cells. In a study by Lim et al., it was demonstrated that the inhibition of Hck resulted in a reduction in Aβ oligomer‐stimulated microglial phagocytosis, thereby confirming the vital role of Hck in microglial neuroprotective function. As for genes involved in neural precursor cell proliferation, it was observed that Notch1 was significantly downregulated in Pld4‐deficient microglia. Notch signaling plays a complex role in remyelination. Zhang et al. reported that the inactivation of Notch signaling in OPCs potentiated the differentiation of OPCs [49]. This may be due to the fact that Notch signaling promotes the expansion of OPCs, but simultaneously suppresses their differentiation [49, 50]. In the current study, Pld4 knockdown significantly reduced the number of Olig2‐positive cells in the corpus callosum of CPZ‐fed mice. This reduction may be due to the downregulated Notch1. However, as Olig2 is an oligodendrocyte lineage marker and cannot distinguish between OPCs and mature oligodendrocytes, further investigation is required to determine the extent to which Notch1 downregulation affects OPC expansion and maturation.

SC79 binds directly to the PH domain of AKT, thereby inducing AKT to adopt a conformation favorable for phosphorylation by upstream kinases [51]. Interestingly, SC79 partially, but not completely, reversed the inhibitory effect of Pld4 siRNA on microglial phagocytosis of myelin debris, whereas SC79 treatment almost completely reversed the impairment of remyelination in Pld4‐deficient mice, suggesting that activation of AKT signaling may promote other processes during remyelination in addition to myelin debris phagocytosis. For example, microglial autophagy, a process that plays a dual role in MS. Autophagy has been postulated to be associated with microglia‐mediated local neuroinflammation. Inhibition of microglial autophagic activation promoted myelin sheath repair in lysophosphatidylcholine‐induced demyelination mice [52]. In contrast, autophagy is also thought to be involved in phagocytosis and clearance of myelin debris, and the impaired autophagy resulted in inadequate clearance of myelin debris and impaired myelination [53, 54]. Since AKT signaling has been demonstrated to regulate microglial autophagy [55], it remains to be investigated whether AKT‐mediated regulation of microglial autophagy is involved in the functions of PLD4 in MS. In addition, SC79 was found to promote the survival of hippocampal neurons in ischemic stroke mice [51]. The above results suggest the neuron‐protective effects of SC79, indicating the potential of SC79 in the application of central nervous system diseases including but not limited to MS.

In summary, we found that Pld4 was increased in the corpus callosum of demyelinated and remyelinated mice. Pld4 deficiency significantly impaired microglial phagocytosis of myelin debris, resulting in impaired remyelination. Mechanistically, Pld4 modulated myelin phagocytosis by microglia via activating of the AKT signaling pathway.

## Ethics Statement

The animal experiment procedure was approved by the Ethics Committee of the Shengjing Hospital of China Medical University and followed the ARRIVE guidelines.

## Conflicts of Interest

The authors declare no conflicts of interest.

## Supporting information


Appendix S1.

## Data Availability

The data that support the findings of this study are available from the corresponding author upon reasonable request.
