# Supplementary material for: Phospholipase D Family Member 4 Regulates Microglial Phagocytosis and Remyelination via the AKT Pathway in a Cuprizone‐Induced Multiple Sclerosis Mouse Model
Source: CNS Neurosci Ther. 2024 Nov 15;30(11):e70111. doi: 10.1111/cns.70111 (PMC11567942; doi:10.1111/cns.70111)

Full unedited blot for Figure 1f

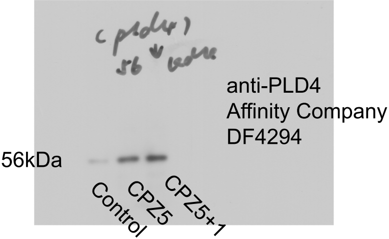

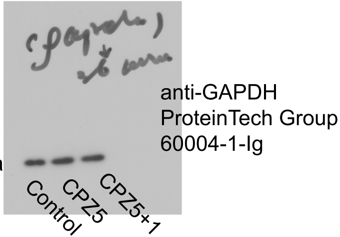

Full unedited blot for Figure 2c

56kDa

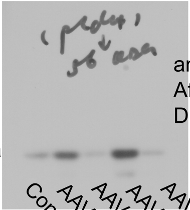

Control
AAV9-shNC
AAV9-shPld4
AAV9-shNC
AAV9-shPld4

anti-PLD4
Affinity Company
DF4294

36kDa

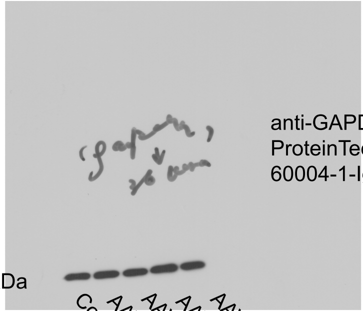

Control
AAV9-shNC
AAV9-shPld4
AAV9-shNC
AAV9-shPld4

anti-GAPDH
ProteinTech Group
60004-1-Ig

Full unedited blot for Figure 4b

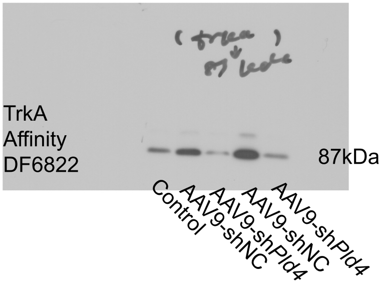

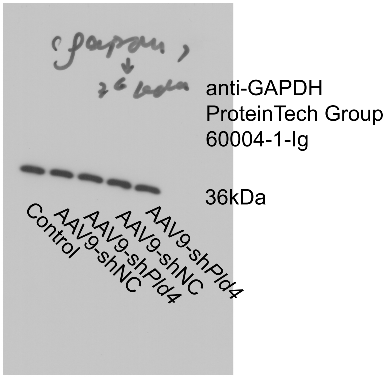

Full unedited blot for Figure 4d

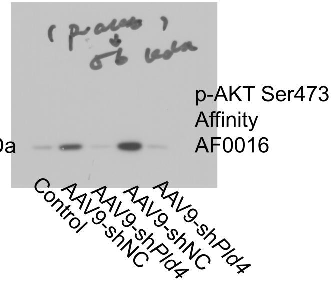

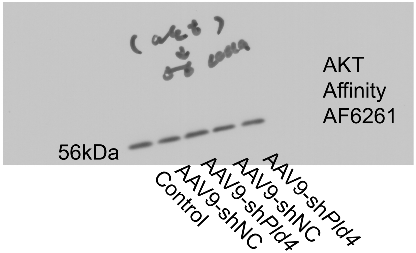

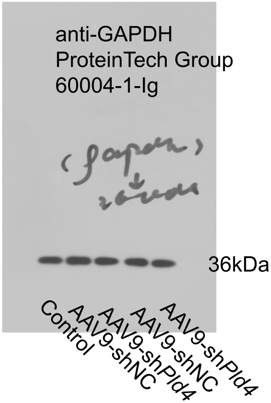

Supplement: Supplementary file 1 — Appendix S1. [file CNS-30-e70111-s001.pdf]
